# Structural analysis of recombinant AAV vector genomes at single-molecule resolution

**DOI:** 10.1371/journal.pone.0339201

**Published:** 2026-07-30

**Authors:** David Rouleau, Dimpal Lata, Serena Dollive, Robert E. Bruccoleri, Laura Van Lieshout, Diane Golebiowski, Ifeyinwa Iwuchukwu

**Affiliations:** 1 Oxford Biomedica (US) LLC, Bedford, Massachusetts, United States of America; 2 Congenomics LLC, Glastonbury, Connecticut, United States of America; Tehran University of Medical Sciences, IRAN, ISLAMIC REPUBLIC OF

## Abstract

Recombinant adeno-associated virus vectors are essential tools for *in vivo* gene therapy, yet heterogeneity in their packaged genomes remains an important safety consideration. To systematically evaluate this heterogeneity, we developed a long-read, read-level analysis pipeline that directly classifies individual AAV genomes and their structural variants from PacBio sequencing data. The workflow combines two components: a tiling step that aligns each read to reference sequences to generate positional patterns, and a parsing step that applies a formal grammar to categorize reads into five structural classes: expected, truncated, snapback, truncated snapback, and others. Each molecule is annotated with strand orientation, breakpoint coordinates, and structural arrangement, enabling precise classification of genome heterogeneity at single-vector resolution. Applied to both single-stranded and self-complementary vector genome preparations, the pipeline achieved high classification accuracy and revealed distinct patterns of genome structure between different vector constructs. In both cases, the majority of genomes were classified as expected full-length species, consistent with the dominant full peaks observed by orthogonal methods. For snapback genomes, breakpoints frequently clustered at discrete sites, with some coinciding with regions predicted to form stable secondary structures and others occurring in less structured regions. This distribution suggests contributions from both sequence-driven folding and additional replication- or processing-related mechanisms. Together, these read-level insights highlight sequence and structural features that shape AAV genome heterogeneity. Importantly, the pipeline demonstrated strong performance in structural classification, maintaining high accuracy even in the presence of sequencing error profiles such as homopolymer-associated indels (insertion or deletion). By integrating structural classification, sequence context, and secondary-structure predictions, our pipeline provides a comprehensive framework for evaluating recombinant adeno-associated virus genome diversity. This approach not only improves resolution of vector genome architecture but also offers actionable insights to guide vector design and production processes for safer and more efficacious recombinant adeno-associated virus therapeutics.

## 1. Introduction

Recombinant adeno-associated vectors (rAAVs) are emerging as a valuable tool for their use in gene therapy, with several candidates showing promising clinical trial outcomes and some already approved by the U.S. Food and Drug Administration [1–3]. At the same time, clinical experience has highlighted important safety concerns including hepatotoxicity, thrombotic microangiopathy, myocarditis, and hepatocellular carcinoma [4–7]. In some high-dose systemic trials, such as those for X-linked myotubular myopathy, patient deaths have been linked to acute liver failure and immune-related complications [8]. More recently, the Elevidys trial for Duchenne muscular dystrophy was temporarily halted after multiple patient deaths, drawing renewed attention to dosing and safety thresholds [9]. Mechanistic studies suggest these outcomes may be driven by innate and adaptive immune responses to AAV capsids and transgene products, off-target biodistribution of vector genomes, and, in rare cases, insertional mutagenesis leading to oncogenic risk [8,10]. These observations emphasize the importance of maintaining the highest possible quality of rAAV vector genomes, as improved potency at lower doses may help reduce immune responses and off-target effects.

Achieving high-quality rAAV preparations, however, remains challenging due to the frequent presence of noncanonical vector genomes, which have been linked to unintended biological effects in vitro and *in vivo* [11]. Another obstacle is the presence of canonical genome structural variation including snapback AAV [12–14], truncated AAV [15,16] and self-priming truncated species [17]. These variants arise during AAV replication and packaging and have been observed both in production samples and *in vivo* [18–20]. These structural variants, despite only containing DNA of the desired vector genome product, can also have a negative impact on vector productivity [21,22]. Thoroughly characterizing the full spectrum of vector genome species in rAAV preparations is therefore essential for both safety and efficacy.

Short-read sequencing methods are limited in their ability to robustly resolve complex structural rearrangements in AAV samples, including truncation products, because they depend on computational reconstruction from fragmented reads [12,19,23]. Recent advances in long-read sequencing technologies, such as single-molecule real-time (SMRT) sequencing, have enabled recovery of high-accuracy AAV genome reads without the need for assembly from short fragments [24]. Using PacBio Sequel II circular consensus sequencing (CCS), we analyzed individual AAV vector genome reads directly, providing a read-level view of the structural variant distribution within the recombinant virus population. This approach gave us a snapshot of the distribution of structural variants present within the recombinant virus population. Since AAV vector characterization is a relatively unique use case, publicly available software that can interpret these full AAV sequences and report structural variant populations are sparse [25,26]. Here we introduce a comprehensive analysis pipeline designed to evaluate the molecular structure of recombinant vector genomes at single-vector resolution. A graphical summary of the pipeline is included in the supplemental file (Fig A in S1 File).

Our pipeline is made up of two programs: tiling and subparsing. Tiling, described previously [27], uses NCBI–BLAST [28] to break sequence reads into a series of alignments that best cover as much of a read as possible. AAV genomes with noncanonical DNA, such as helper plasmid DNA or gene-of-interest plasmid backbone DNA, can be counted in the tiling file as the sequences with BLAST alignments to those sequences. To categorize sequences with only canonical AAV genome alignments, we use the second part of our pipeline, the subparser, which is the focus of this paper.

**Fig 1:**
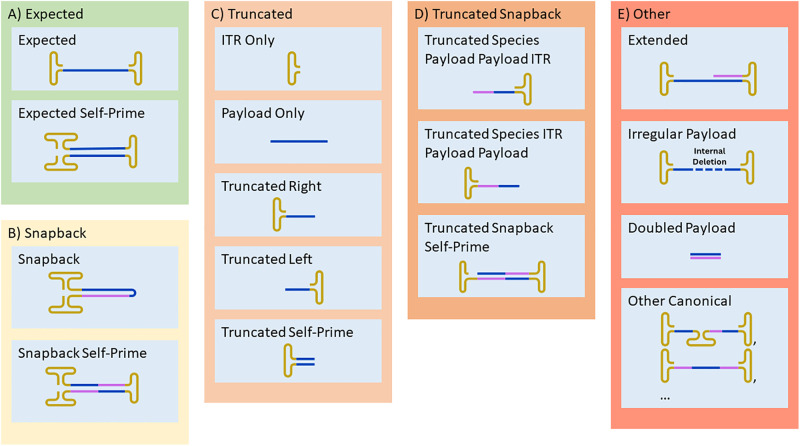
AAV genome structural variants categorized by our subparser program: expected, truncated, snapback, truncated snapback, and other. Gold regions of the vector genome represent ITR sequences, and blue and pink regions represent adjacent non-continuous payload (the AAV’s transgene or gene-of-interest region located between the two ITRs) sequences. A) Expected vector genomes represent the full-length, intact recombinant vector genome, spanning from one inverted terminal repeat (ITR) to the other, and containing the complete payload as designed. B) Snapback genomes are formed when single-stranded AAV DNA folds back on itself via internal complementarity, resulting in partially double-stranded molecules with hairpin-like structures. C) Truncated genomes are incomplete vector genomes that have lost portions of their sequence, typically due to replication or packaging defects [31]. D) Truncated Snapback Genomes are a snapback genome which have also lost portions of their sequence. E) Other vector genomes include miscellaneous structural variants, including extension products resulting from unresolved ITR termination during genome replication [17,32,33]. Four structural variants are labelled as self-priming variants. This different term is used instead of self-complementary for ambiguity since these kinds of structural variants can be present in ssAAV samples due to the DNA repair step of the PacBio SMRT NGS library prep process (Fig B in S1 File).

Our subparser program assigns canonical AAV genome sequences to one of sixteen structural variant categories (Fig 1) which can be grouped into five broader morphological classes: expected, truncated, snapback, truncated snapback, and other genome forms. Several of these categories are labeled as self-priming variants. Self-priming refers to AAV genome molecules in which the 3′ inverted terminal repeat folds back to form a double-stranded hairpin that primes complementary-strand DNA synthesis [29]. We use the term self-priming rather than self-complementary because such structures can also arise during PacBio SMRTbell library preparation, where single-stranded AAV vector may undergo partial strand fill-in reactions that generate molecules resembling a self-complementary vector [30].

The subparser is designed to output sequences with different structural variant classifications into different files so that sequences can be filtered based on their category for further downstream analysis. To further study snapback and truncated structures, which are observed to have variance in AAV preparations [15,32], we developed a custom analysis pipeline to analyze snapback and truncated AAV genome sequence files output by the subparser, identify strand-specific breakpoints in vector genomes, and examine local sequence structure. The full implementation and extension of this method are also described in this paper.

## 2. Materials and methods

### 2.1. AAV vector sample production

The vectors analyzed in this study were recombinant GFP constructs, including a self-complementary GFP (scGFP) containing a genome of approximately 2080 nucleotides (nt) with a payload size of 1831 nt, and a single-stranded GFP (ssGFP) containing a genome of approximately 2162 nt with a payload size of 1872 nt. Both ssAAV and scAAV genomes expressed GFP using the CB promoter (CMV enhancer, chicken beta actin promoter, SV40 intron) and bGH polyA signal. AAV9 vectors were produced using the OXB dual plasmid design as previously described [34] with an adenovirus helper plasmid in suspension VPC 2.0 cells (ThermoFisher). Vectors were purified using POROS AAV9 affinity resin (ThermoFisher) and POROS HQ anion exchange resin.

The vp (vector protein) titer for our vectors were determined by commercially available AAV9-specific ELISA kit (Progen, Heidelberg, Germany, Catalog No. PRAAV9, https://us.progen.com//AAV9-Titration-ELISA/PRAAV9) and performed according to the manufacturer’s instructions. The vg (vector genome) titers were determined using the Bio-Rad ddPCR assay. Droplet generation and reading were performed using the QX200 Droplet Digital PCR System (Bio-Rad Laboratories, Hercules, CA, USA) following the manufacturer’s instructions, specifically using the droplet generation oil and ddPCR Supermix for Probes (No dUTP).

For capsid composition, we performed Capillary Electrophoresis with Sodium Dodecyl Sulfate using Laser-Induced Fluorescence (CE-SDS LIF) under denaturing conditions. Electropherogram data were processed using 32 Karat software. The measured VP ratios were consistent across samples, with VP1:VP2:VP3 ratios of 1.00:1.38:11.48 for SCGFP and 1.00:1.42:11.76 for SSGFP. These ratios are consistent with the canonical VP1:VP2:VP3 stoichiometry for AAV capsids (~1:1:10), indicating proper capsid assembly and composition, and supporting the overall integrity and consistency of the vector preparation. The electropherograms generated from this analysis are included in the supplement (S1 Fig and S2 Fig).

### 2.2. NGS library prep

To assess encapsidated vector genomes, DNA was extracted from enriched AAV fractions using the PureLink™ Viral RNA/DNA Mini Kit (Invitrogen) with an added DNase treatment to remove unencapsidated DNA. The scAAV and ssAAV preparations used in this study had vg/vp ratios of approximately ~0.78 (6.17e13vg/mL/ 7.91e13vp/mL) and ~0.61 (5.64e13vg/mL/ 9.27e13vp/mL) respectively, indicating a high proportion of genome-containing particles.

DNA quality and fragment size distribution were evaluated using an Agilent TapeStation system with the D5000 ScreenTape assay, including D5000 ScreenTape, D5000 sample buffer, and D5000 ladder (Agilent Technologies) according to the manufacturer’s instructions. The TapeStation profiles have been included in the supplemental file (Fig C in S1 File). DNA quality was further assessed using NanoDrop (Thermo Fisher Scientific), and DNA quantification was performed using the Qubit fluorometric assay (Thermo Fisher Scientific).

Samples were normalized before SMRTbell® library preparation to ensure consistency across conditions. SMRTbell® libraries were then prepared according to the PacBio DNA amplicon library preparation protocol (PacBio, Menlo Park, CA), which involved ligation of hairpin adapters to generate circular templates suitable for sequencing. Libraries were purified, quantified, and loaded on the PacBio Sequel II system. Data were initially processed using PacBio SMRT Link v11.1 analysis software. The circular consensus sequence (CCS) calling was performed to generate high-fidelity long reads for downstream structural variant analysis using default parameters from the SMRTLink user guide. No additional user-defined filtering based on read length, genome size, or read quality was applied before analysis. CCS read statistics for these samples have been included in the supplemental file (Table A in S1 File). Sequencing results were analyzed with our NGS structural variant analysis pipeline and our breakpoint analysis program.

### 2.3. AUC analysis

To assess the accuracy of the results of the subparser on real vector genome samples (scGFP and ssGFP), we ran two vector samples through our NGS structural variant analysis pipeline and through Analytical ultracentrifugation (AUC) analysis and compared the results [15,35]. AUC was performed using an Optima AUC instrument (Beckman Coulter) to determine the proportion of full, intermediate, and empty capsids in each AAV sample. Sedimentation profiles were analyzed with the SEDFIT c(s) model, which generates distributions of sedimentation coefficients corresponding to different capsid populations. Integration of the peaks provided the relative percentage of each species within the samples.

### 2.4. Structural variant calling software pipeline

#### 2.4.1. Tiling.

The tiling algorithm, described in detail previously [27], is summarized here for methodological completeness. The tiling algorithm derives its name from its approach of using BLAST to break full sequence reads down into a list of local alignments, the results of which are formatted into space-delimited strings of text called tiles. Each “tile” gives a single BLAST result with the name of the subject sequence that the read segment aligned to, the coordinates of that subject sequence that was aligned to, and the orientation of the alignment. These local alignments are calculated with the read as the query sequence and a list of reference sequences as the subject sequence database. The reference sequences that can be used include the transfection plasmid used to generate the AAV vector product, the host cell genome, and any number of helper plasmids used in vector production.

After many local alignments are generated for a sequence, the tiling algorithm picks up the ordered subset of these alignments that best cover the entire sequence read. The result of this is called a tile pattern, and the occurrence count of each tile pattern within a sample is calculated. The final output of the tiling algorithm, a space separated formatted file labelled as a *.tile.counts file, contains three columns of data for each tile pattern in the sample: the occurrence count, the proportion of reads in the sample that matched the tile pattern, and the tile pattern itself (Table 1). In reasonably homogenous samples with more than 100,000 sequence reads, thousands to tens of thousands of tile patterns may be produced, because the tiling algorithm produces patterns with a nucleotide level resolution. In highly heterogeneous samples, nearly every sequence read may produce a unique tile pattern. Therefore, additional computational analysis is necessary to group similar patterns and classify them into distinct structural variant categories.

**Table 1:** Example tiling algorithm output data.

Count	Proportion	Tile Pattern
100	0.25	ITR-FLIP[1–145](-) Payload[1–1872](-) ITR-FLIP[21–165](+)
90	0.225	ITR-FLIP[21–165](+) Payload[1–1872](-) ITR-FLIP[1–145](+)
80	0.2	ITR-FLIP[1–145](-) Payload[1–1872](-) ITR-FLIP[1–165](-) Payload[1–1872](+) ITR- FLIP[1–145](+)
70	0.175	ITR-FLIP[21–165](+) Payload[1–1872](-) ITR-FLIP[1–165](-) Payload[1–1872](+) ITR- FLIP[21–165](-)
60	0.15	ITR-FLIP[1–145](+) Payload[1–500](+) Payload[1–500](-) ITR-FLIP[21–165](+)

Tiling algorithm output is a tab-separated values (tsv) formatted text file. The first column contains the count of sequences in a sample that matched a single tile pattern. The second column contains that tile pattern’s proportion out of all tiled sequences in the sample. The remaining columns contain the tile pattern itself. Each tile in a tile pattern has three components: a name ascribing the BLAST reference that region aligned to, the coordinates of the BLAST alignment in that reference, and the orientation of the alignment, represented as “ + “ for forward and “-“ for reverse strand. A single ITR reference sequence with the name ITR-FLIP is used for both ITR-FLIP and ITR-FLOP orientation to simplify analysis [36]. Consequently, the tile pattern shown is ITR-FLIP regardless of configuration, but coordinates in range 1–145 indicate ITR-FLIP and 21–165 indicate ITR-FLOP.

#### 2.4.2. Subparsing.

Using the tiling results, we calculated the count of sequences with contaminant DNA in a sequencing run by counting the tile patterns with any tile that isn’t representing an alignment to the AAV reference sequence. We then analyzed sequences consisting exclusively of canonical AAV reference DNA tiles using our subparser program, which was implemented in python version 3.14.2 using the PLY (Python Lex-Yacc) framework (v3.11) [37]. The subparser operates in three main stages: lexing, parsing, and final verification. During the lexing step, the names of each alignment in a tile pattern are converted into a set of tokens, which are a finite number of strings in a context-free language (CFL). During the parsing step, any number of tokens produced by our subparser’s lexer are reduced into a single terminal token using a context-free grammar (CFG). This terminal token will be the tile pattern’s tentative structural variant classification. During the final-check step, the orientation and coordinate data of the tile pattern is checked to ensure the accuracy of the tentative structural variant classification. The final structural variant classification is then assigned to the tile pattern. Our subparser program is available on GitHub.

The conversion of the tile pattern names into a CFL, a set of tokens that are readable by a CFG-utilizing parser, is done by a program called a lexer. The subparser’s lexer converts Payload and ITR-FLIP tile names into “P” and “I” tokens, respectively. The lexer treats both flip and flop ITRs, as well as any other ITR sequence variants, equivalently under the “I” token category to ensure consistent representation across all possible orientations. Single elongated ITRs may be represented by more than one adjacent ITR tile from the tiling program. Consequently, adjacent “I” tokens are reduced to a single “I” token to simplify the CFG and better represent the underlying vector genome structure. An “AND” token is added between each “P” and “I” token to allow for parsing of patterns of any length. These “P”, “I”, and “AND” tokens form the structured CFL input that is passed to the subparser’s parser module for tentative structural variant classification.

**Fig 2:**
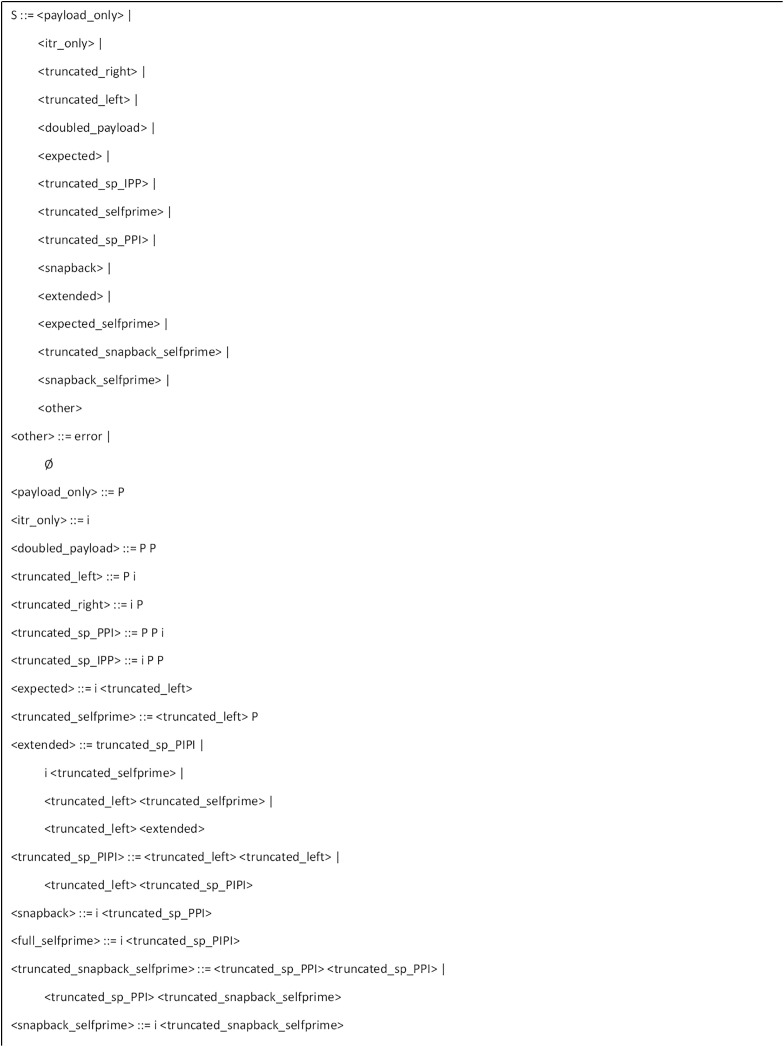
The context-free grammar (CFG) utilized by our subparser program in Backus–Naur form. Tokens in this grammar are”P“,”I” and “AND”. From the tile pattern, tiles that mapped to the reference AAV payload are converted to the”P” token, tiles that mapped to the reference ITRs are converted to the” I” token. An”AND” token is inserted between all adjacent”I” and”P” tokens for right-handed precedence. Tokens within inequality signs are terminal tokens and are accepted as the tile pattern structural variant call if they are the only remaining token.

The reduction of any number of tokens into a single token by following rules in a CFG (Fig 2) is done by a program called a parser. The parser does this by using a left-right, look-ahead (LRLA) parsing algorithm. As the parser proceeds from left to right, the tokens are checked against the rules in the right-hand side of the CFG (Fig 2). If the tokens match a rule, they are reduced into the token on the left-hand side of that rule. The parser treats the “AND” token as an operator with right-hand precedence, and consequently all tokens will be checked together before any reductions occur. This avoids ambiguous CFG rules by giving longer rules precedence over shorter rules. For example, the rule *<truncated_sp_PPI > :: = I AND P AND P* will take precedence over the rule *<truncated_right > :: = I AND P*, since the former would be checked first. Recursive grammar rules such as *<truncated_sp_PIPI > ::= < truncated_left> AND <truncated_sp_PIPI>* allow for parsing theoretically infinitely long, repeating tile patterns. If the parser is unable to reduce the tokens into a single token, then the parser sets the tile pattern’s tentative structural variant designation to *‘other’*.

Since several structural variant calls categorized by our subparser are defined by properties outside of the scope of its parser module’s CFG, including tile orientations and coordinates, a final-check step is done on all tile patterns. For example, an expected structural variant designation necessitates checking the coordinates of the vector genome’s payload sequence to ensure it matches the expected payload sequence. To this end, our subparser checks the tile patterns of sequences given the tentative ‘expected’ structural variant designation to ensure that their payload tile has coordinates matching the expected payload sequence size. If any does not, its structural variant designation is changed to ‘irregular_payload’. The range which a tile pattern’s payload tile start and end coordinates must be in comparison to the reference for it to be considered matching is plus or minus 6 bp by default, but this can be modified by the user on the command line. Another example is the check done for all patterns categorized as ‘snapback’ which validates that snapback payloads originate from opposite strands. A complete list of checks that are made after parsing and the full details of the command line options for our subparser program are both included in the supplemental file (S1 File).

### 2.5. *In silico* data generation

To determine the accuracy of the structural variant calling pipeline, we wrote a sequence generation program that takes as input an AAV genome sequence, a configuration file, and three sets of frequency distributions and outputs a file of simulated AAV sequence reads. For the AAV genome sequence, we designed an AAV genome using publicly available sequence data. Each row of the configuration file gives instructions for one run of the sequence generation program, resulting in one simulated sequence file for each row. These rows denote how to organize payload and ITR sequences in the output file, as to simulate the different structural variants callable by our subparser. For example, one row would give the parameters needed to generate sequences with one ITR then one payload to simulate a truncation product missing its second ITR.

To have these generated sequence files better simulate real long-read sequencing data and to robustly test our structural variant calling pipeline, our sequence generator was designed to introduce up to three different kinds of variation to each sequence generated, depending on applicability. To simulate the error rates of different long read sequencers, we generated four different sequence files with a chance of each generated base written being incorrect at a rate of 0, 0.001, 0.01 and 0.05 for each reduction rule in our subparser’s CFG [38–43]. To simulate homopolymer and snapback variations seen in real long read sequencing data [14,44], we used the three input frequency distribution sets calculated from real PacBio SMRT NGS sequencing runs. The first set was a single distribution of the frequency of a mutation for each homopolymer size. The second set of frequency distributions was the frequency of each indel size for each homopolymer size. The last set was a single frequency distribution of different lengths of the two payload sequences in a snapback, normalized to reference payload size. The sequence generation program, the generated sequence files, and the frequency distributions used are available on GitHub. Further information on our *in silico* data generation program is included in the supplemental file (S1 File).

### 2.6. Breakpoint-associated structure predictions in snapback and truncation events

To better understand the structural basis for vector genome heterogeneity, particularly within snapback and truncation structural variants, we developed a custom Python–R-based pipeline that performs breakpoint detection followed by local RNA secondary structure analysis. This pipeline operates on subparser output files, which contain sequence-level tile patterns representing the arrangement of vector components such as ITRs and payloads along with their positions and strand orientations.

We parsed these tile patterns using regular expressions to identify genomic breakpoint positions where the continuity of payload segments is interrupted. For snapback structures, we defined the breakpoints as the 3′ nucleotide position in the 5′ payload tile where two payload segments appear in opposite orientations and are flanked on both ends by ITRs (Fig D in S1 File). For self-priming truncation structures, where a single ITR is flanked by two reverse-complementing partial payloads, breakpoints were defined as positions where the flanking payloads were truncated during incomplete replication (Fig E in S1 File). For snapback and self-priming truncation structures, all breakpoint positions were mapped and reported separately for the plus and minus strands.

To investigate the potential structural context of observed breakpoints, we extracted sequence windows surrounding each breakpoint from the reference payload sequence. A range of window sizes was used to capture secondary structure features. Each sequence fragment was folded using RNAfold (v2.5.1, ViennaRNA package [45] with the parameters -p -d2 --noLP to compute the minimum free energy (MFE) and centroid structures. For higher reliability, we retained only those breakpoint-associated windows in which the MFE and centroid structures were identical, indicating consistency in the predicted folding. In cases where multiple overlapping structures were present for the same breakpoint, the structure window with the most negative MFE was selected as the representative conformation for downstream analysis. The final output of this analysis included the breakpoint position, associated sequence window, and corresponding MFE values. To visualize these results, strand-separated plots were generated using ggplot2 package in R [46], showing both the frequency of breakpoints and their associated structural stability across the genome. This approach enabled a comparative view of folding potential around different structural variant breakpoints within AAV vector preparations.

To further assess sequence features at breakpoint sites, we applied a chi-square goodness-of-fit test using the chi-square from the *SciPy* statistical package (v1.9.3) implemented in Python (v3.10.12). This analysis compared observed nucleotide frequencies at breakpoints positions to the background base composition of the reference payload sequence. Analyses were performed separately for the plus and minus strands.

## 3. Results

### 3.1. *In silico* testing

To validate the accuracy of our structural variant calling pipeline, we used our own sequence generator program to produce 68 files of 10,000 AAV genome structural variants sequences and ran them through our structural variant calling pipeline. The files consisted of four replicates of 17 structural variant sequence files, each with a different level of error applied to its sequences. We ran these files through our structural variant calling pipeline and complied the subparser results into a single data table with one row for each file comparing the percentage of sequences in each file that matched its expected structural variant category (S1 Table). We then calculated the average and standard deviation of sequences that matched their expected structural variant category across all 68 files. We then did this same calculation for different subsets of files to investigate pipeline accuracy when different variations were applied to the generated sequences (Table 2).

**Table 2:** *In silico* subparsing summary statistics.

File Subset	Average Match Percentage	Standard Deviation
**All Files**	99.37%	0.56%
**Error Rate 0**	99.51%	0.39%
**Error Rate 0.001**	99.51%	0.41%
**Error Rate 0.01**	99.45%	0.44%
**Error Rate 0.05**	99.01%	0.80%
**Snapbacks**	99.10%	0.58%
**Self-Primes**	98.76%	0.77%

For all files, the average percentage of sequences in each file that matched their expected structural variant was 99.40% with a standard deviation of 0.56%. For the sequence generation error rates of 0, 0.001, 0.01 and 0.05, the average match percentage and standard deviation across all structural variants were 99.51% and 0.39%, 99.51% and 0.41%, 99.45% and 0.44%, and 99.01% and 0.80% respectively. The average match percentage across all snapback structural variant sequence files which had randomized snapback breakpoint sites was 99.10% with a standard deviation of 0.58%. The average match percentage across all self-priming sequence files was 98.76% with a standard deviation of 0.77%.96.79% for the self-priming expected sequence file with an error rate of 5%. The average match percentage across all self-priming sequence files was 98.76% with a standard deviation of 0.77%.

The minimum match percentage was 96.79% for the self-priming expected sequence file with an error rate of 5%, which is expected due to how we generated homopolymer indels. This was expected because 1) whether or not an indel was generated on a homopolymer was randomized based on frequency distributions, 2) the length generated indels was randomized based on frequency distributions, 3) longer sequences have more homopolymers, and 4) uncommon large indels seen in our frequency distributions prevent BLAST alignment in the tiling step. Consequently, longer sequences like self-priming sequences are more likely to not match their expected category when generated by the sequence generation program.

### 3.2. *In vitro* testing

To evaluate the consistency and improved resolution of our updated pipeline, we compared the subparser’s output to AUC results for two different AAV samples (Table D in S1 File). For both vector types, the overwhelming majority of genomes were classified as expected full-length species, in close agreement with the full capsid proportions measured by AUC (94.5% vs. 94.8% for scGFP and 88.6% vs. 85.8% for ssGFP). Only small fractions of reads were assigned to noncanonical forms such as snapback (≈2% in both samples) and truncated genomes (1.7% in scGFP and 4.4% in ssGFP), with “truncated snapback” and “other canonical” species each below 1%.

Reads classified as non-VG and unclassified sequences were also very low (<1% for scGFP and ≈3–0.6% for ssGFP). Together, these data show that the subparser’s structural classification produced results highly consistent with the independent AUC measurements, confirming that the pipeline can accurately resolve the major and minor genome populations in rAAV samples.

Non-VG sequences were further categorized using *AWK* to count non-VG database sequences aligned to by the tiling algorithm. Such categories included sequences with helper DNA, sequences with GOI backbone DNA, sequences with hcDNA, and chimeric sequences, which had any combination of these non-VG alignments. For both samples, only backbone aligned sequences had a frequency greater than ~0.1%, with a frequency of 0.72% for the scGFP sample and 2.92% for the ssGFP sample.

### 3.3. Breakpoint-associated structure predictions

**Fig 3:**
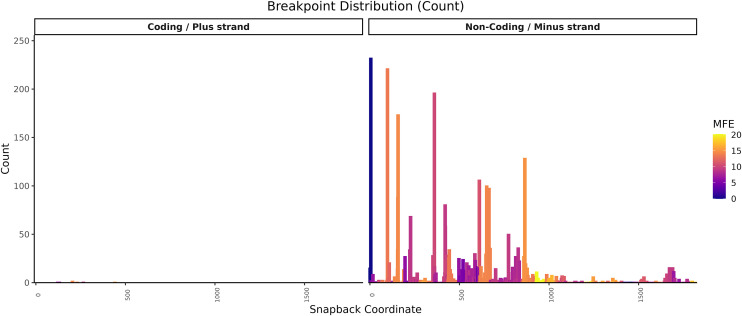
Distribution of snapback breakpoints with local structural stability estimates in scGFP.

**Fig 4:**
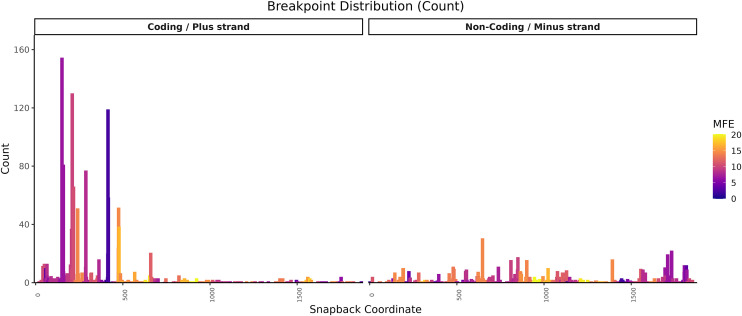
Distribution of snapback breakpoints with local structural stability estimates in ssGFP.

Snapback breakpoints identified in both scGFP and ssGFP preparations localized to discrete sites along the genome rather than being randomly distributed (Fig 3 and Fig 4). For scGFP, snapback breakpoints were highly concentrated on the minus strand, particularly downstream of the promoter and within the GFP payload region, with several positions showing strong enrichment. In contrast, the plus strand contained relatively few, lower-frequency breakpoints. For ssGFP, the overall number of breakpoints was lower than in scGFP, but the breakpoints still occurred at defined sites rather than being broadly dispersed.

Bar plots show the frequency of breakpoint positions across the AAV payload sequence on the plus (coding) and minus (non-coding) strands. The x-axis indicates nucleotide position within the payload, and the y-axis indicates the number of reads with a breakpoint at each position. Each bar represents a single nucleotide breakpoint site and is colored according to the absolute value of the minimum free energy (MFE) of the predicted local RNA secondary structure surrounding that breakpoint, with yellow indicating more stable predicted structures. In scGFP, recurrent breakpoint hotspots were observed near positions ~98, 157, 360, 611–665, and 865.

Bar plots show the frequency of breakpoint positions across the AAV payload sequence on the plus (coding) and minus (non-coding) strands. The x-axis indicates nucleotide position within the payload, and the y-axis indicates the number of reads with a breakpoint at each position. Each bar represents a single nucleotide breakpoint site and is colored according to the absolute value of the minimum free energy (MFE) of the predicted local RNA secondary structure surrounding that breakpoint, with yellow indicating more stable predicted structures. in ssGFP, recurrent breakpoints were broadly distributed, including positions ~151, 210, 415, 476, 647, 850, 1392, and 1709–1732

In scGFP, snapback breakpoint hotspots were observed approximately at positions 98, 157, 360, 611–665, and 865. These recurrent hotspots were predominantly localized within non-coding/minus-strand regions. Several of these coincided with regions of moderate to high predicted local structural stability. However, the strongest hotspot, near position 5, occurred in a region with minimal predicted structure, and other high-frequency sites were also observed in regions with only moderate folding potential. These breakpoints mapped across multiple functional regions, including the EGFP coding sequence and regions near the SV40 intron.

In ssGFP, recurrent snapback breakpoints were more broadly distributed approximately at different positions including 151, 210, 415, 476, 647, 850, 1392, and 1709–1732. Several enriched breakpoint sites within coding/plus-strand regions, including positions near 476, were associated with regions of moderate to high predicted local structural stability. In contrast, other recurrent breakpoints occurring in both coding/plus-strand and non-coding/minus-strand regions were associated with relatively low predicted folding potential. Notably, strong breakpoint enrichment near positions 415 and 417 was observed despite minimal predicted local structural stability. Additional hotspots within non-coding/minus-strand regions, including positions near 1709–1732, were also associated with only low to moderate folding potential. Mapping shows that these breakpoints span coding and intron-associated regions. Overall, snapback breakpoint hotspots were observed in regions with both high and low predicted local structural stability. Detailed breakpoint positions, counts, and corresponding MFE values are provided as Supplementary Table Files (S2 Table and S3 Table).

In addition to snapbacks, we examined truncation-associated self-priming events in both scGFP and ssGFP (Fig F and G in S1 File). For scGFP, truncation breakpoints were observed on both strands, with a higher frequency on the minus strand. Several sites clustered at defined positions, whereas others were more broadly distributed across the genome. Some of these breakpoints coincided with stable predicted secondary structures, while many occurred in regions without strong folding potential. A subset of low-frequency breakpoints, particularly those with an even genomic distribution, may reflect random DNA shearing introduced during SMRTbell library preparation rather than genuine replication-derived events. For ssGFP, truncation-associated breakpoints were fewer in number and appeared more dispersed than in scGFP. The relatively low counts and broader distribution suggest that truncation events in ssGFP are less strongly dictated by specific sequence or structure motifs. Detailed truncation breakpoint positions, counts and corresponding MFE values are provided as supplementary tables (S4 Table and S5 Table).

To integrate these findings, we tested whether sequence composition itself biases breakpoint formation. Nucleotide composition at breakpoints deviated significantly from background expectations. For snapback genomes, scGFP minus-strand breakpoints were enriched for T (χ² test, p = 8.5 × 10 ^−^ ⁸⁸), whereas the plus strand showed no significant enrichment (p > 0.05). In contrast, ssGFP plus-strand breakpoints were enriched for G and T (χ² test, p = 1.6 × 10 ^−^ ⁷⁰), while the minus strand showed a weaker deviation (p = 3.9 × 10 ^−^ ⁴). In truncation-associated self-priming events, scGFP showed strand-specific bias, with C enrichment on the plus strand (χ² test, p = 7 × 10 ^−^ ⁶) and no significant enrichment on the minus strand (p = 0.14). By contrast, ssGFP truncations showed no strong enrichment on either strand (p > 0.05).

## 4. Discussion

Adeno-associated viral vector (AAV) genome heterogeneity remains a critical concern in gene therapy vector design and quality control. While previous studies have explored the diversity of packaged genomes through various methods, including short-read NGS, they have been unable to reveal the complete structure of entire packaged vector genomes within an AAV sample [47].

To evaluate the robustness and accuracy of our pipeline, we conducted *in silico* benchmarking experiments. The subparser results on the *in silico* data shows that the pipeline was robust in its classifications in the presence of simulated sequencing error rates, homopolymer indel rates, and random snapback breakpoints. Even though classification accuracy declined slightly at the highest simulated error rates and with self-priming sequences, it still correctly classified more than 96% of reads. In the presence of high simulated error rates and random snapback breakpoints, the average match percentage remained over 97%. These findings support the robustness of the grammar-based approach to the modeled artifacts included in our simulations, while also recognizing that misclassification may still occur for structures or error profiles not represented in the simulation design. Accordingly, these results increase confidence that many differences observed in real AAV samples are likely to reflect biological heterogeneity rather than sequencing noise, although this conclusion remains bounded by the scope of the simulation framework.

The reduction in accurate calls by our subparser on the *in silico* self-priming sequences was likely due to how our sequence generator program was designed to simulate homopolymer-associated indel errors observed in real sequencing results. This is supported by the lowest match percentage being in the expected self-priming sequence files, which also required payloads that mostly matched the size of the reference AAV genome’s payload sequence. Since these payloads were more likely to contain indels, it was more likely that these sequences failed this check. Even with this expected payload check, with the possibility of large indels being generated, and with a simulated error rate of 5%, the subparser still accurately categorized 96.79% of generated sequences. These results support the subparser’s ability to correctly classify sequences in the presence of indel sequencing errors seen in NGS data.

To evaluate our pipeline’s capabilities on real sequencing data, we performed library prep on two vector samples with different genome structures and analyzed the resulting data. Although a formal minimum read depth threshold was not established, our analyses indicate that datasets with a productive ZMW fraction (P1) above ~50% provide sufficient coverage for reliable sequence analysis. This is consistent with PacBio guidelines for optimal sequencing performance [48]. For both the scGFP sample data and the ssGFP sample data, the subparser had an expected sequence percent comparable to the AUC assay’s full mass peak. Small differences between expected peak size and AUC full mass peak could be a result of non-canonical vector genome structural variants with sizes similar to the expected vector genome size. Despite these differences, the correlation between the AUC results and our NGS pipeline results demonstrates that the pipeline accurately classifies long-read sequencing data. The observed differences in structural variant frequencies between scAAV and ssAAV likely reflect inherent variation in genome structure and packaging. While sequencing-related effects may also contribute, the concordance between the full and partial frequencies across the AUC and NGS suggests the genome variation is authentic. While AUC provides robust, orthogonal estimates of populations (e.g., full, partial, and empty capsids), it does not reveal sequence-level information. In particular, AUC cannot localize breakpoints, report strand orientation, identify sequence context or secondary-structure features, or distinguish among different canonical structural variants that share similar mass. Our read-level approach fills these gaps by linking each structural call to exact genomic coordinates and sequence features.

One limitation of this study is that the ssGFP vector genome analyzed here is approximately 2.2 kb, which is substantially smaller than the typical AAV packaging capacity. Since genome size can influence packaging outcomes and genome heterogeneity, the structural variant distributions observed in this sample may not fully reflect those present in larger vectors closer to the packaging limit. Future studies using vectors spanning a broader range of genome sizes will help further evaluate the impact of genome design on structural variant formation.

Beyond classification, the output of the subparser was analyzed further to investigate biological insights into the structural variant formation process. Our breakpoint analysis provides important insights into the mechanisms underlying snapback and truncation variant formation in AAV genomes. In both scGFP and ssGFP samples, snapback breakpoints were not randomly distributed but instead clustered at discrete genomic sites. Several of these positions overlapped with regions predicted to form stable local secondary structures (low MFE), suggesting that structural folding may promote or stabilize snapback formation. However, other high-frequency breakpoints showed only weak folding potential, indicating that additional processes such as replication stress or non-homologous end joining (NHEJ) may also contribute. Interestingly, similar nonrandom clustering of snapback breakpoints and nucleotide enrichment patterns was previously described [15], where G/C nucleotide enrichment at major breakpoints was identified across multiple AAV vector genomes. Our results extend these findings by showing that, in scGFP and ssGFP samples, the specific enrichment pattern varies, suggesting that the preferred nucleotide composition at breakpoints may depend on vector-specific sequence context.

In contrast, truncation events displayed a more uniform and dispersed distribution. The truncation breakpoints were fewer and appeared more scattered across the genome, consistent with a more stochastic origin. These patterns suggest that truncation products may arise largely from random fragmentation or replication-related instability. Notably, by mapping breakpoints and examining their nearby genomic context, our pipeline can highlight features such as promoters or repetitive elements that may help explain structural variant formation. Furthermore, the pipeline can also be used for the estimation of the sizes of snapback and truncated genomes directly from read-level data, offering insight into the prevalence of shorter genome fragments. In wild-type AAV, such shorter species have been reported to play regulatory roles, whereas in recombinant AAV gene therapy vectors, they are best regarded as heterogeneous by products or contaminants. Overall, our findings suggest that secondary structure is one contributing factor but not the only one in the formation of structural variants in AAV vectors. High-resolution structural variant classification and strand-aware breakpoint analysis provides a framework for understanding the diversity of vector genome forms and offers a path to optimizing vector design, helper components, and production conditions.

Although this study utilized PacBio Sequel II data, the analysis pipeline is based on high-accuracy circular consensus sequence (CCS) reads and is therefore not inherently platform-specific. Nevertheless, differences in sequencing chemistry, throughput, and read-length distributions between earlier and more recent PacBio instruments (e.g., Sequel, Sequel IIe, and Revio) may influence data characteristics. These factors could affect the detection and relative representation of certain structural variant classes, particularly low-abundance or structurally complex genomes. Newer platforms with improved accuracy and higher throughput may enhance sensitivity for rare variants. While the core tiling and grammar-based classification approach is expected to remain robust across platforms, systematic cross-platform comparisons would be valuable to further assess potential biases and confirm the generalizability of the method.

## 5. Conclusions

Developing long-read sequencing technologies has given researchers the ability to fully sequence vector genomes. Although numerous structural variants have been reported in the literature, there remains a limited number of bioinformatics workflows specifically designed to comprehensively analyze rAAV genome heterogeneity. Using *in silico* and real world sequencing data and by comparing our pipeline results to AUC results, we have demonstrated that our structural variant calling pipeline can accomplish comprehensive characterization of rAAV vectors robustly and accurately. Additionally, the tile patterns that match each structural variant can be written into separate files so that downstream analysis can be restricted to only sequences with a desired morphology. To complement structural variant calling and to demonstrate analysis of sequences filtered by our subparser program, we also developed a structural analysis module that examines strand-specific breakpoints and local RNA folding potential. This adds mechanistic insight into how sequence structure may influence genome heterogeneity, especially in snapback and truncation variants.

A limitation of the current framework is that the grammar-based subparser was designed for the classification of canonical AAV genome architectures only. Extending the grammar to include Rep/Cap-, helper-, and backbone-derived structures, including ITR-linked helper forms relevant to replication-competent particles, would broaden the applicability of the method and is an important future direction. Understanding the industry’s need for such analysis [25], we are actively working towards completing such an extension using the grammar-based methodology described.

Overall, this pipeline advances the resolution at which vector genome complexity can be studied and understood. Its application across ssAAV and scAAV samples and its correlation with AUC results demonstrates its practical utility for both research and vector quality control.

## Supporting information

S1 File Supplemental information, figures, and tables. (DOCX)

S1 Fig scGFP CE electropherogram. (PDF)

S2 Fig ssGFP CE electropherogram. (PDF)

S1 Table *In silico* subparsing results. (XLSX)

S2 Table Snapback scGFP results. (CSV)

S3 Table Snapback scGFP results. (CSV)

S4 Table Truncation self-priming scGFP results. (CSV)

S5 Table Truncation self-priming ssGFP results. (CSV)
