# Supplementary material for: Structural analysis of recombinant AAV vector genomes at single-molecule resolution
Source: PLoS One. 2026 Jul 30;21(7):e0339201. doi: 10.1371/journal.pone.0339201 (PMC13422874; doi:10.1371/journal.pone.0339201)
Supplement: S1 File — (DOCX) [file pone.0339201.s001.docx]

**Figure A: GRAPHICAL SUMMARY OF THE STRUCTURAL VARIANT CALLING PIPELINE**

**
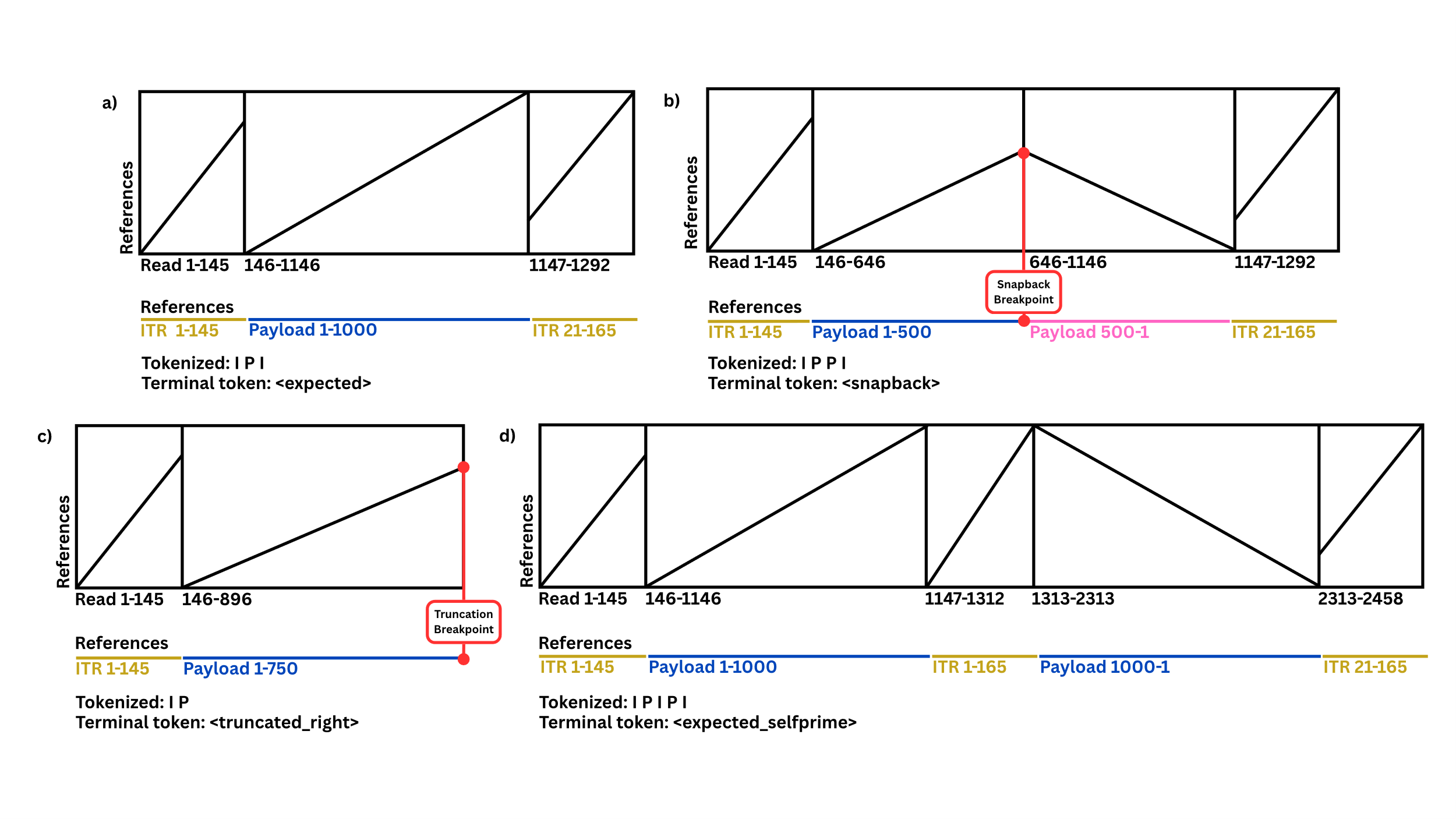
**

A graphical summary of the structural variant calling pipeline for a) the expected vector genome b) a snapback vector genome c) a truncated vector genome missing its right ITR and d) a self-priming expected vector genome. The alignment dot-plots show the result of tiling, with the x-axis corresponding to base pairs of a sequence read and the y-axis corresponding to the read’s alignment to different reference sequences. The tiling algorithm uses BLAST to make these alignments. For more information on tiling, see https://www.biorxiv.org/content/10.1101/2025.07.25.666743v1.full.

The x-axis shows which coordinates of the read aligned to the corresponding reference sequences and their coordinates below each alignment dot-plot. A single ITR reference sequence with the name ITR-FLIP is used for both ITR-FLIP and ITR-FLOP orientation to simplify analysis. Consequently, the aligned reference sequence shown is ITR-FLIP regardless of configuration, and coordinates in range 1-145 indicate ITR-FLIP and 21-165 indicate ITR-FLOP. A range of 1-165 indicates an extended ITR sequence. Here the payload reference sequence is set as 1000bp for simplicity.

The tiling algorithm outputs the concatenated alignment data to the subparsing program. The subparsing program then tokenizes these references. Example results of this are shown beneath each plot as the “Tokenized” text. The subparsing algorithm then uses a context-free grammar to determine if the tokenized string matches any structural variants. The result is a terminal token, shown as the “Terminal token” text beneath each plot. Breakpoints for the snapback vector genome and truncated vector genome are also annotated, showing where the snapback folded back on itself and where the truncated vector genome was truncated respectively.

**Figure B: LIBRARY PREP SECOND-STRAND SYNTHESIS MECHANISM**


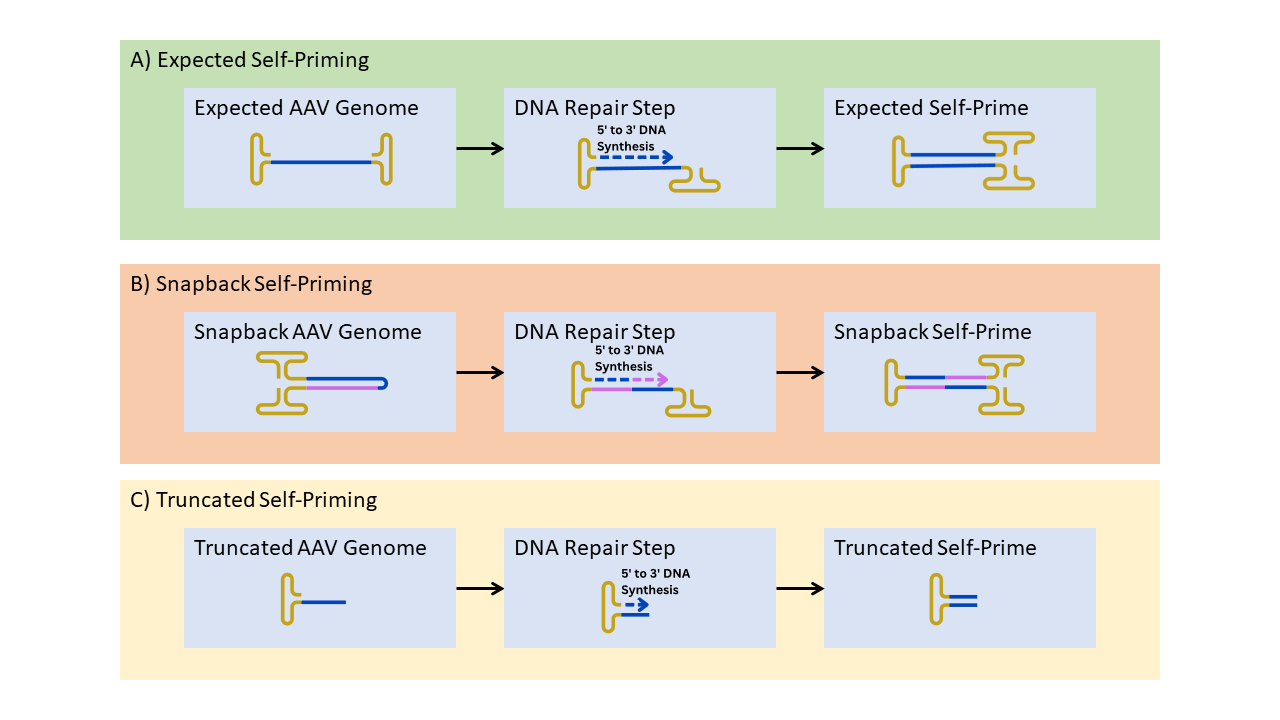
The possible synthesis of second strands during the DNA repair step of SMRT NGS library prep on single stranded AAV genomes results in self-priming vector genomes. This can occur for A) expected B) snapback and C) truncated vector genomes.

**Figure C: TapeStation analysis of extracted vector genomes prior to library prep**


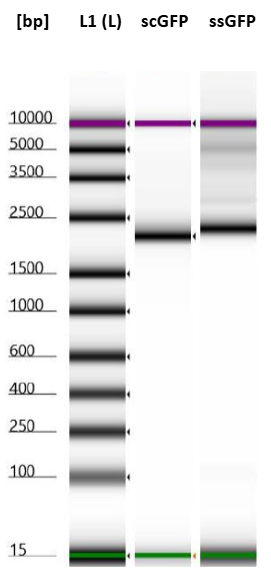


Extracted genomes from the scGFP and ssGFP AAV preparations were analyzed by TapeStation before PacBio sequencing. For the scGFP sample, the predominant TapeStation peak was observed near the expected monomer genome size (~2.1 kb), whereas CCS sequencing identified a dominant population of approximately 4 kb molecules. As described previously in the supplemental file figure B, self-priming events can occur during PacBio SMRTbell library preparation, resulting in sequencing reads that contain both the vector genome and its reverse-complement sequence within a single molecule. Consistent with this mechanism, a large fraction of scGFP reads were classified as expected_selfprime by our NGS analysis pipeline. TapeStation and long-read sequencing, therefore, provide complementary measurements of vector genome structure and may report different apparent genome lengths for self-primed molecules.

**Table A: CCS QC statistics for the scGFP and ssGFP samples**

| Vector | CCS Reads | Mean Length (bp) | Median Length (bp) | Max Length (bp) | Mean Q |
| --- | --- | --- | --- | --- | --- |
| scGFP | 250,512 | 3,976 | 4,073 | 10,199 | 78.7 |
| ssGFP | 162,022 | 2,493 | 2,165 | 9,999 | 80.6 |

CCS read statistics are provided as sequencing quality metrics, including read count, read length, and read quality. The scGFP sample yielded 250,512 CCS reads with a mean read length of 3,976 bp (median 4,073 bp) and a mean quality score of Q78.7. The ssGFP sample yielded 162,022 CCS reads with a mean read length of 2,493 bp (median 2,165 bp) and a mean quality score of Q80.6.

**SUBPARSER FINAL-CHECK RECLASSIFICATIONS**

The Subparser program does three logical checks after parsing since coordinate and orientation data are out of the scope of its context-free grammar. First, if a pattern classified as ‘expected’ or ‘expected_full’ has payload coordinates that don’t match the expected vector genome coordinates, then its structural variant classification is changed to ‘irregular_payload’. Second, if a pattern classified as ‘snapback’, or ‘snapback_selfprime’ has only adjacent payload tiles with the same orientation, its classification is changed to ‘irregular_payload’. If the pattern has a combination of same and different orientation adjacent payloads, its classification is changed to ‘other’. Lastly, if a pattern is classified as ‘truncated’_sp_IPP’ (truncated species ITR Payload Payload), ‘truncated_sp_PPI’ (truncated species Payload Payload ITR) or ‘truncated_snapback_selfprime’, and any of its payloads are the same orientation, then its classification is changed to ‘other’.

**SUBPARSER COMMAND LINE OPTIONS**

Our subparser program is run via the command-line using three required arguments and nine optional arguments. The required arguments are the input file, output directory, and the expected size of the payload sequence.

The first three optional arguments allow the user to modify how the program handles different types of optional input. The first optional input is the summary file generated by the tiling algorithm for each tiling file. If the corresponding argument is raised, the program will automatically look for the summary file in the directory of the input file. The second type of optional inputs are the tile patterns in the input file that contain noncanonical alignments, such as to a helper plasmid. If this flag is not raised, all such sequences will be ignored from classification and frequency calculations. If the flag is raised, all such sequences will be classified as `other`. The third type of optional inputs are homopolymer tiles in the patterns of the input file. If the corresponding flag is raised all sequences with these tiles will be classified as `other`.

The second three arguments allow the user to modify the programs output. The first of these allows the user to modify how the program groups structural variants together, or to disable grouping completely. The second determines whether the program will output a counts file for each found structural variant containing all tile patterns that were given that classification. The last of these enables debugging output files from the ply library showing detailed information on the parser’s CFG.

The last three optional arguments allow the user to determine the strictness with which our subparser program will classify sequences as `expected`, or matching the desired vector sequence, along with the required payload size argument. The first of these flags toggles whether coordinates are checked for payload sequences. If the flag is raised, any tile pattern matching the structure of the reference genome is called expected regardless of its coordinates. This flag is disabled by default. The argument allows the user to modify how close the coordinates of a payload sequence need to be to the reference payload size to be considered matching. By default this value is set to 6. The last of these flags toggles whether or not the program should raise an error if the proportion of analyzed sequence that match the expected reference drops below 50%. By default, this flag is disabled.

**SUBPARSER OUTPUT FORMAT**

The output format of our subparser program is a tsv file with a summary containing frequency data for all structural variants found (Table A) followed by tables with detailed information on the sequences for each classification (Table B). Our subparser program also automatically outputs a seaborn bar plot and Matplotlib pie plot for quickly visualizing the summary table.

**Table B: SUBPARSER SUMMARY TABLE OUTPUT**

| Bin | Sequences | Proportion | Patterns | Percent Full |
| --- | --- | --- | --- | --- |
| expected | 208236.5 | 0.956635 | 8554 | 100.00% |
| snapback | 4333.5 | 0.019908 | 3413 | 6.28% |
| truncated | 3834 | 0.017613 | 3186 | 0.97% |
| other | 1243 | 0.00571 | 1302 | 65.53% |
| truncated_snapback | 29 | 0.000133 | 32 | 3.45% |
| Totals | 217676 | 1 | 16487 |  |

Data displayed are the results of the scGFP sample. Percent Full column displays what percentage of sequences with that structural variant contain at least one full payload. Structural variants were binned into five groups as displayed in figure 1 of the manuscript. Payloads were considered a match to the reference if they were within 6bps of the reference payload size at both the start and end coordinates.

Structural variant frequencies were determined from sequence reads after using the tiling algorithm [https://doi.org/10.1101/2025.07.25.666743]. Sequences from the same zero-mode waveguide (ZMW) were consolidated before parsing by the tiling algorithm. This table includes only sequences classified as vg by the tiling algorithm; sequences classified as non-vg or left unclassified are excluded.

**Table C: SUBPARSER DETAILED TABLE OUTPUT EXAMPLE**

| Category | Sequence Count | Repeats | Proportion of expected_selfprime | Linearity | Contains Irregular ITRs | Contains PolyX | Contains a Full Payload | Tokenized | Tile Pattern |
| --- | --- | --- | --- | --- | --- | --- | --- | --- | --- |
| Expected selfprime | 18341 | 1 | 0.08882 | non_linear | FALSE | FALSE | TRUE | I P I P I | ITR-FLIP[1-145](-)  Payload[1-1831](-)  ITR-FLIP[25-141](-)  Payload[1-1831](+)  ITR-FLIP[1-145](+) |
| Expected selfprime | 17841 | 1 | 0.086399 | non_linear | FALSE | FALSE | TRUE | I P I P I | ITR-FLIP[1-145](-)  Payload[1-1831](-)  ITR-FLIP[25-141](+)  Payload[1-1831](+)  ITR-FLIP[1-145](+) |
| Expected selfprime | 17296 | 1 | 0.08376 | non_linear | FALSE | FALSE | TRUE | I P I P I | ITR-FLIP[21-165](+)  Payload[1-1831](-)  ITR-FLIP[25-141](-)  Payload[1-1831](+)  ITR-FLIP[21-165](-) |

The detailed structural variant information table output by our subparser program. Data displayed are the top three most common tile patterns of the scGFP sample.

**DETAILED *IN SILICO* SEQUENCE GENERATION DESCRIPTION**

To robustly test our subparser program, we simulated the error rates seen in real long read sequencing. This was done by generating four replicates of each generated sequencing file, each with a different simulated error rate. The error rate would give the frequency at which the sequence generation program would add the wrong base when writing a sequence. Error rates used were 0.001 to simulate the sequencing error rate after circular consensus sequencing calling from the PacBio Sequel IIe, and 0.01 and 0.05 to simulate the different error rates reported for Nanopore long read sequencing. We also used an error rate of 0 to determine classification profiles of the *in silico* data when no error rate was applied.

Homopolymer mutation rate was introduced to simulate increased incidence of insertions or deletions (InDels) during long-read sequencing of homopolymers. To implement this, we used frequency distributions calculated from real PacBio Sequel II data (Supplementary Table Files 1 and 2: HomopolymerIncidenceRates.xlsx and HomopolymerSizeDistributions.csv). First, the sequence generator would decide whether to introduce an InDel using the homopolymer InDel frequency distribution, containing the frequency of InDels for each homopolymer size. A random float would be generated in the range from 0 to 1, inclusive, and if it was less than the frequency of InDels for that homopolymer size, then an InDel would be generated. For example, our distribution has an InDel incidence frequency of ~0.04 for a homopolymer of size 4. If the random float generated is less than 0.04, then the sequence generator would add an InDel to the generated homopolymer. To determine what size of InDel to generate, the sequence generator would utilize our set of InDel type frequency distributions calculated from real PacBio Sequel II data. The InDel type frequency distribution for the homopolymer being written would be converted to a cumulative distribution, then a random float would be generated. The first value that the random float is less than in the cumulative distribution determines which InDel will be generated.

Snapback breakpoint variation was introduced to simulate the random breakpoints seen in snapback AAV genome structural variants. This variation was introduced to all snapback and truncated snapback structural variants. This was implemented with a normalized snapback size frequency distribution derived from real sequence data generated on a PacBio Sequel II (Supplemental Table File 3: SnapbackSizeDistribution.xlsx). When any snapback was generated by the sequence generator script, the size and orientations of both adjacent payload sequences was randomly pulled from the snapback size frequency distribution in the same way InDels were pulled from the homopolymer InDel frequency distribution set and rescaled to the expected reference vector’s payload size.

**Figure D:** **BREAKPOINT OF SNAPBACK STRUCTURE.**


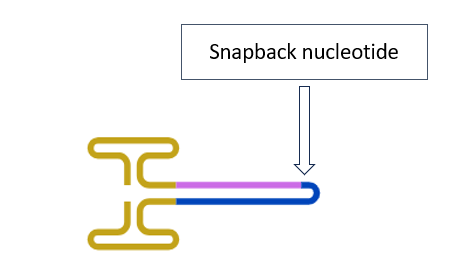


**Table D: DATA COMPARISON BETWEEN OUR SUBPARSER PROGRAM UTILIZING A CFG AND ORTHOGONAL AUC DATA FOR SCAAV AND SSAAV SAMPLES**

| Vector Sample Type | Classification | Percentage | AUC Peak | Percentage |
| --- | --- | --- | --- | --- |
| scGFP | expected | 94.52% | Full | 94.81% |
| scGFP | snapback | 1.97% | Other | 5.19% |
| scGFP | truncated | 1.74% |  |  |
| scGFP | truncated_snapback | 0.01% |  |  |
| scGFP | other canonical | 0.56% |  |  |
| scGFP | helper | 0.03% |  |  |
| scGFP | backbone | 0.72% |  |  |
| scGFP | hcDNA | 0.02% |  |  |
| scGFP | chimera | 0.04% |  |  |
| scGFP | unclassified | 0.39% |  |  |
| scGFP | Total | 100.00% | Total | 100.00% |
| scGFP | Total non-VG | 0.81% |  |  |
| ssGFP | expected | 88.59% | Full | 85.79% |
| ssGFP | truncated | 4.38% | Other | 14.21% |
| ssGFP | snapback | 2.50% |  |  |
| ssGFP | truncated_snapback | 0.00% |  |  |
| ssGFP | other canonical | 0.79% |  |  |
| ssGFP | helper | 0.03% |  |  |
| ssGFP | backbone | 2.92% |  |  |
| ssGFP | hcDNA | 0.04% |  |  |
| ssGFP | chimera | 0.14% |  |  |
| ssGFP | unclassified | 0.59% |  |  |
| ssGFP | Total | 100.00% | Total | 100.00% |
| ssGFP | Total non-VG | 3.14% |  |  |

AUC data is renormalized to exclude empty capsids. Total non-VG was calculated as a sum of sequences which had any tile in their tile pattern with an alignment to the helper, backbone, or hcDNA sequences, as well as any combination of such sequences (chimera). Unclassified sequences were calculated as the difference between a fasta file’s sequence count and the sum of the first column of its tiling algorithm output. NGS data was normalized to total tile patterns plus untiled sequences.

**Figure E:** Breakpoint of Truncation self-priming structure.


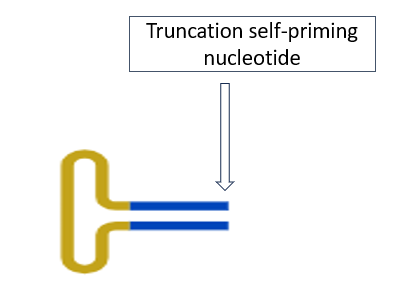


**Figure F:**  Distribution of truncation breakpoints with local structural stability estimates in scGFP


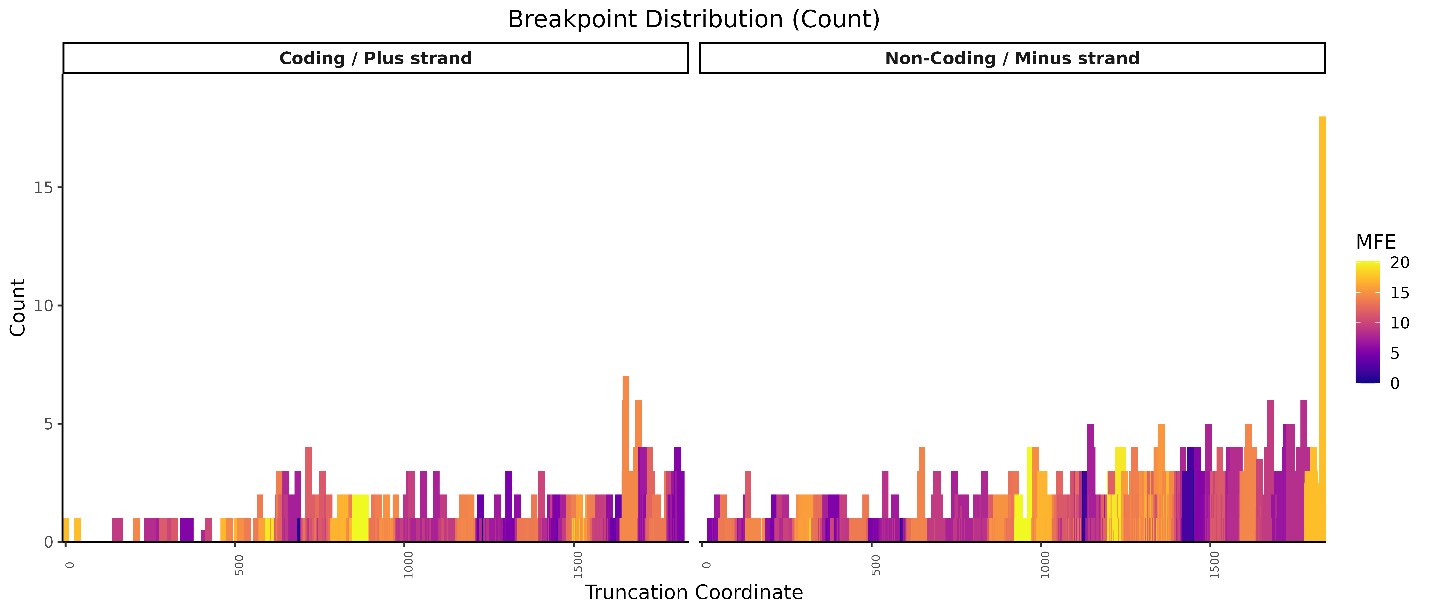


Bar plots display the count of breakpoints across the AAV payload sequence for plus (coding) and minus (non-coding) strands. The x-axis shows breakpoint positions, and the y-axis indicates the count of reads at each position. Each bar represents a single nucleotide position. Bars are colored according to the absolute value of the minimum free energy (MFE) of the predicted RNA secondary structure around each breakpoint, with yellow indicating more stable structures.

**Figure G:** Distribution of truncation breakpoints with local structural stability estimates in ssGFP


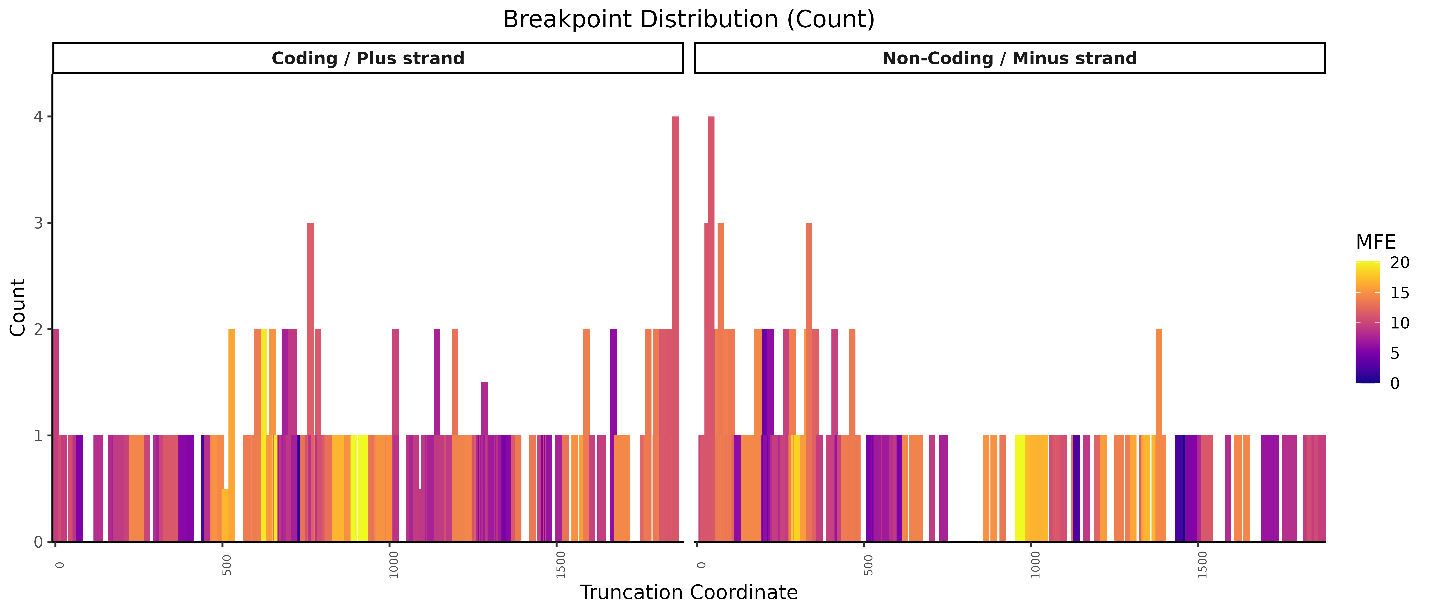


Bar plots display the count of breakpoints across the AAV payload sequence for plus (coding) and minus (non-coding) strands. The x-axis shows breakpoint positions, and the y-axis indicates the count of reads at each position. Each bar represents a single nucleotide position. Bars are colored according to the absolute value of the minimum free energy (MFE) of the predicted RNA secondary structure around each breakpoint, with yellow indicating more stable structures.
