# Supplementary material for: Structural analysis of recombinant AAV vector genomes at single-molecule resolution
Source: PLoS One. 2026 Jul 30;21(7):e0339201. doi: 10.1371/journal.pone.0339201 (PMC13422874; doi:10.1371/journal.pone.0339201)
Supplement: S1 Fig — (PDF) [file pone.0339201.s002.pdf]

32Karat

ProteomeLab PA 800 System - Carbohydrate Analysis Report

Electropherogram trace:

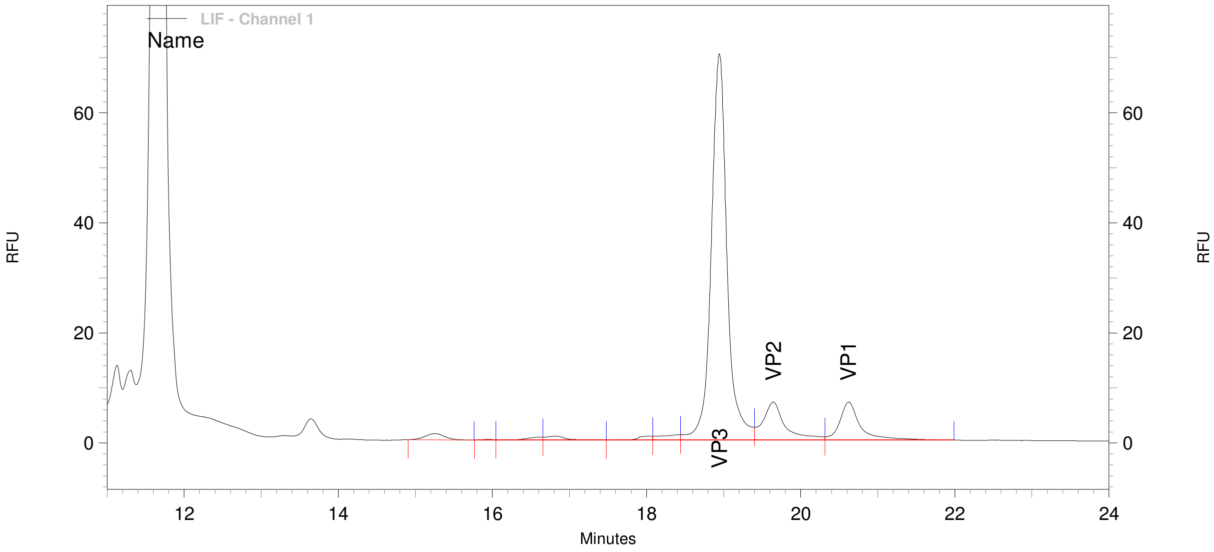

LIF - Channel 1 Results

Name	Migration Time	Corrected Area	Corrected Area Percent
	15.250	87027.978	1.864
	15.933	5662.929	0.121
	16.617	32871.775	0.704
	16.817	53970.307	1.156
	18.025	35736.366	0.765
	18.425	64240.760	1.376
VP3	18.946	3496489.395	74.896
VP2	19.646	472379.317	10.118
VP1	20.621	420099.026	8.999
Totals		4668477.854	100.000
