# Supplementary material for: Structural analysis of recombinant AAV vector genomes at single-molecule resolution
Source: PLoS One. 2026 Jul 30;21(7):e0339201. doi: 10.1371/journal.pone.0339201 (PMC13422874; doi:10.1371/journal.pone.0339201)
Supplement: S2 Fig — (PDF) [file pone.0339201.s003.pdf]

32Karat

ProteomeLab PA 800 System - Carbohydrate Analysis Report

Electropherogram trace:

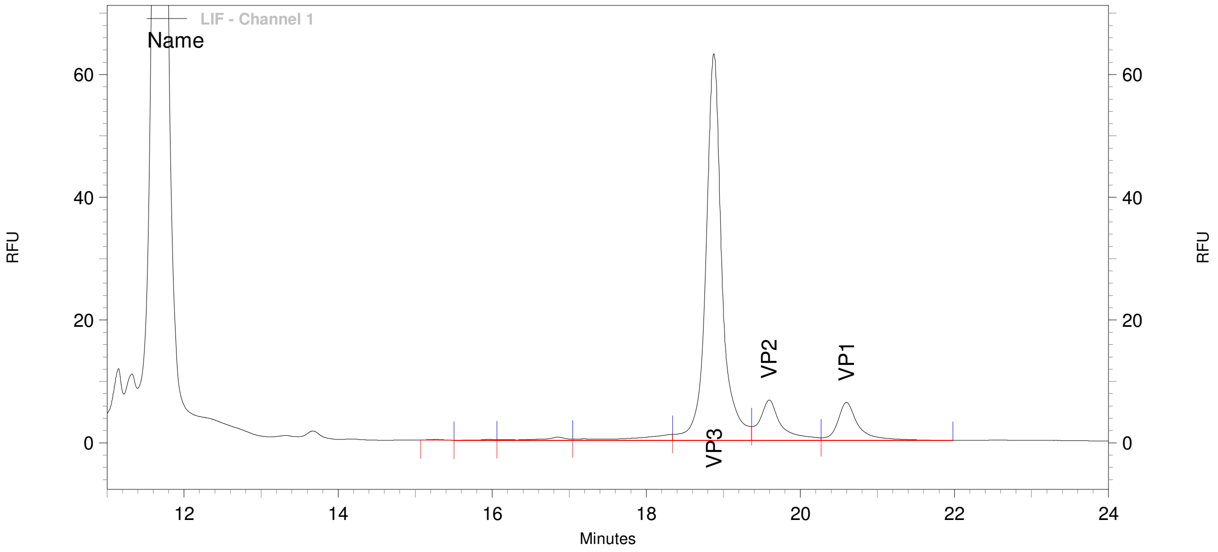

LIF - Channel 1 Results

Name	Migration Time	Corrected Area	Corrected Area Percent
	15.275	5692.962	0.136
	15.954	7424.476	0.177
	16.837	45713.596	1.093
	18.342	109577.683	2.619
VP3	18.879	3203662.026	76.584
VP2	19.596	435361.837	10.407
VP1	20.600	375757.638	8.983
Totals		4183190.218	100.000
